# Correlation between Corneal Volume and Corneal Biomechanics and Corneal Volume Significance in Staging and Diagnosing Keratoconus

**DOI:** 10.1155/2024/8422747

**Published:** 2024-05-29

**Authors:** Zhiqing Wu, Yaohua Zhang, Yong Li, Fang Yang, Xirui Su, Yan Gao, Shengsheng Wei, Jing Li

**Affiliations:** ^1^Department of Ophthalmology, Hospital of Shaanxi Normal University, Xi'an 710004, China; ^2^Shaanxi Eye Hospital, Xi'an People's Hospital (Xi'an Fourth Hospital), Affiliated People's Hospital of Northwest University, Xi'an 710004, China; ^3^Department of Ophthalmology, Renmin Hospital, Hubei University of Medicine, Shiyan 442000, Hubei, China

## Abstract

**Purpose:**

To investigate the relationship between corneal volume (CV) at different zones and corneal biomechanics in keratoconus (KC) along with the significance of CV in diagnosing and staging KC.

**Methods:**

This prospective clinical study included 456 keratoconic eyes (Group B) and 198 normal eyes (Group A). Using the topographic KC classification method, Group B was divided into subgroups based on severity (mild, moderate, and severe). The CVs of the 3 mm, 5 mm, and 7 mm zones and biomechanical parameters were obtained by Pentacam and Corvis ST. The diagnostic utility of multirange CVs at different disease stages and severity was determined using a receiver operating characteristic (ROC) curve analysis.

**Results:**

The CV of the 7-mm zone had the strongest correlation with A1V, A2T, PD, DA ratio max (2 mm), DA ratio max (1 mm), ARTh, integrated radius, SPA1, and CBI (*p* < 0.01). The CVs of the Group B subgroups were significantly lower than those of Group A for each diameter range (*p* < 0.05). There were significant differences between the severe, mild, and moderate subgroups for the 3 mm zone (*p* < 0.05, all). The 3 mm zone CV exhibited better diagnostic ability in each group for distinguishing KC from the normal cornea (Groups A vs. B: area under the ROC curve (AUC) = 0.926, Groups A vs. B1: AUC = 0.894, Groups A vs. B2: AUC = 0.925, Groups A vs. B3: AUC = 0.953).

**Conclusion:**

The CV significantly decreased in keratoconic eyes. Progressive thinning in the 3 mm zone may be a valuable measurement for detecting and staging KC. Combining the CV examination with corneal biomechanical information may effectively enhance the ability to detect KC.

## 1. Introduction

Keratoconus (KC) is a noninflammatory form of corneal ectasia characterized by progressive thinning, steepening, and apical conic protrusion of the cornea that onsets at puberty [1]. The KC apex is displaced inferiorly, inducing irregular myopic astigmatism and causing gradual vision impairment [1, 2]. The most characteristic changes in KC are those affecting the overall morphology and structure of its tissue and biomechanics, which directly reflect severity. KC management depends mainly on disease stage and patient visual requirements. As the condition worsens, common treatments, such as spectacles and rigid contact lenses, have shifted to surgical procedures, and corneal grafting is the traditional treatment for advanced KC [3]. KC has been reported to be the reason of 18% penetrating keratoplasty and 40% deep anterior lamellar keratoplasty [4, 5].

KC detection remains an area of significant interest. Currently, the primary diagnostic and classification criteria for KC are based on anterior surface curvature data of the cornea obtained by corneal topography, which provides two-dimensional imaging of the corneal surface [6]. Corneal tomography is a three-dimensional imaging technique [7] that has been shown to be critical for enhancing the sensitivity and specificity of detecting corneal ectasia compared with corneal topography [8]. Pallikaris et al. [9] reported that corneal volume (CV) can be a predictive factor for the development of corneal ectasia after refractive surgery and may be considered to avoid post-laser-assisted in situ keratomileusis ectasia. Pentacam (Oculus, Dutenhofen, Germany), a relatively new three-dimensional analyzer equipped with a rotating Scheimpflug camera, allows reliable CV assessment [10]. CV as a structural feature of the cornea affects corneal biomechanics and differs from corneal thickness [11]. It reflects corneal topographical and pachymetric alterations with a single value and may detect rare anomalies that are difficult to detect by corneal topography or/and corneal central thickness (CCT) evaluation [12].

Corvis ST (OCULUS Optikgeräte GmbH, Wetzlar, Germany) is a noncontact biomechanical measurement device that mainly focuses on reflecting comprehensive corneal biomechanical properties [13]. The updated Corvis ST software has produced unique parameters related to corneal deformation and stiffness in vivo [13–15]. Studies have demonstrated that these new parameters are highly efficient in diagnosing KC [16–18]. CV is a structural characteristic that contributes to the biomechanical profile of the cornea. Sedaghat et al. [19] found that corneal hysteresis and corneal resistance were correlated with CV and that CV was valuable for determining patient qualification and may be used to predict the need for refractive surgery. In this study, we evaluated the potential correlation between CV and biomechanical parameters of keratoconic corneas provided by Corvis ST. To the best of our knowledge, this study is the first to compare the CV values of different KC stages to those of normal corneas at different diameter areas and explore the grading and diagnostic utility of CV values for KC.

## 2. Materials and Methods

In this cross-sectional study, 395 patients with keratoconic eyes (456 eyes, Group B) and 99 participants with normal eyes (198 eyes, Group A) who visited the Ophthalmology Department of Xi'an People's Hospital (Xi'an Fourth Hospital) between January 2016 and December 2023 were recruited. All patients provided informed consent. The study protocols were approved by the Institutional Review Board and complied with the tenets of the Declaration of Helsinki.

The inclusion criteria of Group A were binocular anterior surface curvature <46.5 D, posterior surface curvature <57.2 D, and thinnest corneal thickness (TCT) >490 *μ*m.

For Group B, KC was diagnosed based on the Rabinowitz [20] criteria. The KC grading method was based on the topographic KC (TKC) classification method provided by Pentacam [21, 22]. Group B was further divided into the subgroups Group B1, 110 eyes (TKC = 1 or 1–2, mild KC); Group B2, 206 eyes (TKC = 2 or 2–3, moderate KC); and Group B3, 140 eyes (TKC = 3, 3–4, or 4, severe KC).

In order to avoid age and gender bias, the participants from Group A were matched to those from Group B for age and sex. None of the participants had a family history of KC.

The exclusion criteria in this study included significant corneal scarring or associated ocular pathology, ocular allergies, nystagmus, previous ocular surgery or trauma, systemic disease, diabetes, or connective tissue disease.

All patients underwent comprehensive ophthalmic examinations, and biomechanical parameters were measured and analyzed by Corvis ST. Corvis ST is a novel biomechanical analyzer developed in a noncontact mode using a released air puff. Video footage of compression deformation was obtained using a high-speed Scheimpflug camera. Approximately 140 cross-sectional images of the cornea were recorded over a collimated air puff for 30 ms. Biomechanical parameters were obtained at the end of this process using the built-in software.

Pentacam is a commonly used corneal tomographic image analysis instrument. It uses a Scheimpflug camera to scan from the anterior surface of the cornea to the posterior surface of the lens and obtains the morphological parameters of the anterior segment by analyzing the collected data. The CV data were obtained from the Pentacam.

All examinations were performed by trained technicians in the same examination room. Only measurements designated as “OK” quality specifications were considered valid.

### 2.1. Statistical Analyses

Data analysis was performed using the SPSS statistical software (ver. 22.0 for Windows; IBM Corp., Armonk, NY, USA). The normal distribution of the parameters was checked using the Kolmogorov–Smirnov test. Data following a normal distribution: after control for CCT, a partial correlation analysis was performed to determine the correlation between CV and biomechanical parameters; a one-way analysis of variance (ANOVA) with post-hoc Bonferroni analysis was used to compare the CV values between Groups A and B; otherwise, they were compared by the nonparametric Kruskal–Wallis test. The Bonferroni test and post-hoc test for Kruskal–Wallis analysis were used for pairwise comparisons. A receiver operating characteristic (ROC) curve analysis was performed to determine the diagnostic accuracy of the CV values in distinguishing keratoconic eyes from normal eyes. The best cut-off points were set to be the maximum values of sensitivity (%) + specificity (%)-1. Statistical significance was set at a *p* < 0.05.

## 3. Results

A total of 456 keratoconic eyes from 395 patients and 198 normal eyes from 99 matched control patients were included in this study.  Table 1 summarizes the corneal biomechanical parameters obtained using Corvis ST. The correlation between CV and corneal biomechanical parameters is presented in Table 2.

The mean ± standard deviation CV for the 3 mm, 5 mm, and 7 mm diameter zones for keratoconic eyes was 3.44 ± 0.52, 10.38 ± 0.63, and 22.81 ± 1.38 mm^3^, respectively. There were strong correlations between the CV and biomechanical parameters for 5 mm and 7 mm diameter ranges. The 5 mm zone CV and 7 mm zone CV were strongly correlated with A1V, A2T, A2V, DA ratio max (2 mm), DA ratio max (1 mm), ARTh, integrated radius, SPA1, and CBI (*p* < 0.01). In addition, CV of the 7 mm zone was strongly correlated with PD, HCDA (*p* < 0.01).

Significant differences in CV for multiple ranges were detected between the subgroups of Group B (*p* < 0.05) (Table 3). Differences were found between each subgroup and Group A (*p* < 0.05) and between the severe, mild, and moderate subgroups for the 3 mm zone (*p* < 0.05).

**Figure 1:**
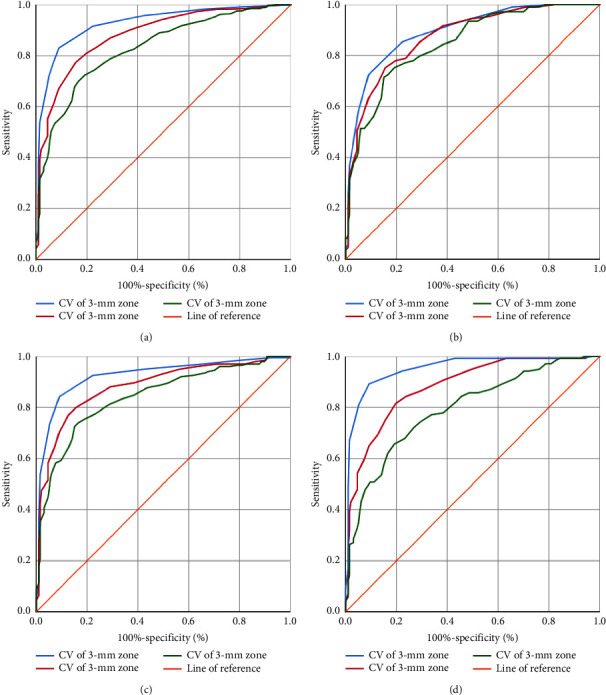
Receiver operating characteristic (ROC) curves of the different corneal volumes (CVs) at the 3 mm, 5 mm, and 7 mm zones. (a): Group A (*n* = 198) versus Group B (*n* = 456), (b): Group A (*n* = 198) versus Group B1 (*n* = 110), (c): Group A (*n* = 198) versus Group B2 (*n* = 206), and (d): Group A (*n* = 198) versus Group B3 (*n* = 140).

Figures 1(a)–1(d) and Tables 4–7 present the results of the KC ROC curve analysis. The area under the curve (AUC) values ranged from good to excellent for all measured parameters. The ROC curves and AUC values showed a strong ability to discriminate between the CVs of the 3 mm, 5 mm, and 7 mm zones. The 3 mm zone CV had better sensitivity and specificity for discriminating between normal and keratoconic eyes than the other zones.

## 4. Discussion

Several studies have shown a relationship between corneal resistance factor and corneal hysteresis derived from the ocular response analyzer and CV [19, 23]. However, it has been demonstrated that the repeatability of the ORA is low. The Corvis ST is a novel developed tool for measuring corneal deformation in a noncontact mode by a released air puff (air-puff diameter 3.05 mm) with acceptable reliability [24, 25]. Due to the update of the Corvis ST software, new biomechanical parameters have been incorporated, such as the DA ratio max (2 mm), integrated radius, ARTh, SPA1, and CBI. Several studies have shown that these new parameters are highly efficient in diagnosing KC [16–18]. CV reflects topographical and pachymetric changes and characterizes corneal morphometric changes with a single value [12]. After the control for CCT, significant correlations were found between most of the biometric parameters evaluated and the CVs of the 7 and 5 mm zones. This confirms the relevance of the biometric and volumetric profile of the cornea in the measurement of biomechanics in KC. This implies that, among patients with KC, decreased CV values may indicate compromised corneal deformation. The progressive increase in corneal irregularities and a decrease in corneal CV might underlie the correlations between CV and biomechanical parameters.

The human cornea is a heterogeneous, viscoelastic biological material. The tissue response to the application of a force depends not only on the magnitude of that force but also on the force's velocity [26]. KC deformation is easier with weaker matrix collagen fibers and a thinner cornea. KC progression leads to the destruction of the corneal stroma, causing instability of the corneal biomechanical properties and weakened mechanical strength [27].

SP A1 is a parameter that reflects corneal rigidity. It is defined as the ratio of the pressure loading (imposed by the air-puff) on the cornea to the displacement of the corneal apex (from the undeformed state to the first applanation). The SP-A1 value has been reported to be lower in thin corneas than in normal corneas [28]. Molecular biology studies have showed that enzyme activation plays a key role in the degradation of the corneal stroma and in corneal thinning, thus affecting corneal stiffness [8]. The CBI is an integration of several dynamic corneal response parameters measured by the Corvis ST (consists of A1V, DA ratio (2 mm), ARTh, SP A1, and integrated radius), reflecting a comprehensive corneal biomechanical property [28]. ARTh is a parameter of the Ambrósio relational thickness to the horizontal profile [13]. Since CCT is generally assumed to be a parameter that fluctuates in parallel with CV. The CV of the 7 mm zone had the strongest correlation with biomechanical parameters. It is consisted with the previous reports [19, 23].

Each corneal layer has been reported to undergo histopathological changes in KC, which are more pronounced in the central area than in the peripheral cornea. Corneas with KC have a reduction in the number of lamellae, particularly in cone development regions, without breaks in the anterior limiting lamina or scarring [29]. Furthermore, it has been proposed that collagen lamellae expand in relation to the cone protrusions [30]. Ectasia and thinning in KC are associated with lamellar breakage into multiple bundles of collagen fibrils and the loss of anterior lamellae. These structural changes may occur in addition to the lateral shifting of lamellae due to the pressure gradient over the cornea and provide a potential explanation for the central thinning of the mass, ultimately leading to a reduction in stromal thickness [31]. The CV reduction in the keratoconic eye is due to corneal thinning, which typically occurs in the central and paracentral cornea [32]. In addition, sliding of the corneal collagen matrix may also contribute to CV loss in subclinical or initial-stage KC [27]. There were significant differences in CVs between Groups A and B. Additionally, there were significant differences between the severe, mild, and moderate subgroups for the 3 mm zone. These results demonstrated that CV could be used to distinguish KC from normal eyes and indicate KC severity.

Progressive corneal thinning is a well-known indicator of KC progression. Keratometric and corneal volumetric alterations have recently been reported to be more prominent in patients with subclinical KC than in those with forme fruste KC [33]. Corneal thinning and CV loss are characteristic alterations observed in eyes with subclinical KC [34]. Ambrósio et al. [35] demonstrated that the percentage of increase in volume distribution was significantly altered in mild-to-moderate keratoconic eyes. Toprak et al. [34] calculated volumes using radius increases in steps of 0.05 mm and found that an anterior apex-centered CV value at 1.0 mm significantly contributed to distinguish subclinical KC from normal. Corneal thinning typically occurs in the central or paracentral cornea [32], and nipples or oval cones in the central or paracentral cornea are most common [3]. In our study, the 3 mm zone CV had a good-to-excellent AUC value for discriminating between normal and mild-to-severe keratoconic eyes.

Identification of subclinical or mild stage of KC in patients with few clinical signs is challenging. Reports recommend using CV as an additional measurement to avoid corneal ectasia or reduce the risk of ring-segment extrusion in the implantation of Intacs [23, 30, 35]. In this study, the 3 mm zone CV had the highest diagnostic utility and could be included as a way of classifying KC type and severity and monitoring the progression of this condition. The combination of CV and various biomechanical parameters may provide more complete information regarding KC grading, diagnosis, and treatment from both the morphological and biomechanical perspectives. This study mainly discussed the diagnostic value of CV in patients with KC and did not involve the diagnosis of suspicious KC. Hence, a larger sample size and stricter data screening are needed.

## 5. Conclusions

In conclusion, among patients with KC, decreased CV values may indicate compromised corneal deformation. CV is correlated with the biomechanical properties of keratoconic eyes. The CV of the 3 mm zone has an acceptable diagnostic accuracy for KC detection and progression. The combination of CV and biomechanical analyses could enhance the ability to detect corneal ectasia and even to predict patients' outcomes.

## Figures and Tables

**Table 1:** Corneal biomechanical parameters obtained with Corvis ST.

Parameters	

A1T	First applanation time
A1V	The first velocity of applanation
A2T	Second applanation time
A2V	The second velocity of applanation
PD	Peak distance (width or bending distance)
Radius	Central curvature radius at the highest concavity
HCDA	Deformation amplitude of the highest concavity
A1 DfL	Deflection length of the first applanation
A2 DfL	Deflection length of the second applanation
DA ratio max (2 mm)	The maximal value of the ratio between the deformation amplitude at the apex and at 2 mm from the corneal apex
DA ratio max (1 mm)	The maximal value of the ratio between the deformation amplitude at the apex and at 1 mm from the corneal apex
ARTh	Ambrósio relational thickness to the horizontal profile = Pachy (thinnest)/pachy progression
Integrated radius	Inverse of the radius of curvature during the concave phase of the deformation
SP A1	Stiffness parameter at the first applanation = (Adj AP1 − bIOP)/A1 deflection amplitude
CBI	Corvis biomechanical index = EXP (beta)/(1 + EXP (beta)) (consists of A1V, DA ratio (2 mm), ARTh, SP A1, and integrated radius)

Adj AP1: adjusted pressure at A1; bIOP: biomechanically corrected intraocular pressure.

**Table 2:** Relationships between corneal volume (CV) and biomechanical parameters for different corneal diameters of Group B.

Parameters	CV of 3 mm zone		CV of 5 mm zone		CV of 7 mm zone	
	*R*	*p*	*r*	*p*	*r*	*p*
A1T	0.022	0.649	−0.093	0.052	−0.085	0.078
A1V	0.086	0.073	0.294	<0.001^*∗∗*^	0.297	<0.001^*∗∗*^
A2T	0.041	0.390	0.140	0.003^*∗∗*^	0.167	<0.001^*∗∗*^
A2V	−0.046	0.342	−0.300	<0.001^*∗∗*^	−0.289	<0.001^*∗∗*^
PD	0.010	0.831	−0.123	0.010^*∗*^	−0.151	0.002^*∗∗*^
Radius	0.003	0.956	−0.065	0.178	−0.081	0.092
HCDA	−0.017	0.725	0.313	<0.001^*∗∗*^	0.304	<0.001^*∗∗*^
A1 DfL	0.004	0.927	0.106	0.027^*∗*^	0.096	0.045^*∗*^
A2 DfL	−0.019	0.690	−0.006	0.908	0.029	0.540
DA ratio max (2 mm)	0.087	0.069	0.294	<0.001^*∗∗*^	0.337	<0.001^*∗∗*^
DA ratio max (1 mm)	0.060	0.208	0.389	<0.001^*∗∗*^	0.398	<0.001^*∗∗*^
ARTh	−0.048	0.314	−0.541	<0.001^*∗∗*^	−0.548	<0.001^*∗∗*^
Integrate radius	0.048	0.318	0.484	<0.001^*∗∗*^	0.503	<0.001^*∗∗*^
SP A1	0.008	0.860	−0.266	<0.001^*∗∗*^	−0.285	<0.001^*∗∗*^
CBI	−0.014	0.764	0.156	0.001^*∗∗*^	0.164	0.001^*∗∗*^

^*∗*^: *p* < 0.05, ^*∗∗*^: *p* < 0.01.

**Table 3:** Differences in corneal volume (CV) at different diameter ranges by disease severity.

	Group A	Group B1	Group B2	Group B3	*F*	*p*
CV of 3 mm zone	3.88 ± 0.20	3.50 ± 0.23^a^	3.47 ± 0.74^a^	3.36 ± 0.23^abc^	44.706	<0.001
CV of 5 mm zone	11.37 ± 0.57	10.43 ± 0.62^a^	10.34 ± 0.65^a^	10.39 ± 0.61^a^	120.556	<0.001
CV of 7 mm zone	24.52 ± 1.21	22.72 ± 1.30^a^	22.67 ± 1.48^a^	22.93 ± 1.17^a^	82.743	<0.001

^a^: Significant difference to group A; ^b^: significant difference to group B1; ^c^: significant difference to group B2.

**Table 4:** Area under the curve (AUC) and cut-off values of corneal volume (CV) for the 3 mm, 5 mm, and 7 mm zones for distinguishing Group A (*n* = 198) from Group B (*n* = 456).

	AUC	SE	*p*	Cut-off value (D)	Sensitivity (%)	Specificity (%)
CV of 3 mm zone	0.926	0.011	<0.001	0.739	83.0	90.9
CV of 5 mm zone	0.881	0.014	<0.001	0.616	77.3	84.3
CV of 7 mm zone	0.828	0.017	<0.001	0.533	70.0	83.3

**Table 5:** Area under the curve (AUC) and cut-off values of corneal volume (CV) for the 3 mm, 5 mm, and 7 mm zones for distinguishing Group A (*n* = 198) from Group B1 (*n* = 110).

	AUC	SE	*p*	Cut-off value (D)	Sensitivity (%)	Specificity (%)
CV of 3 mm zone	0.894	0.019	<0.001	0.634	72.5	90.9
CV of 5 mm zone	0.873	0.020	<0.001	0.560	85.3	70.7
CV of 7 mm zone	0.847	0.022	<0.001	0.560	75.2	80.8

**Table 6:** Area under the curve (AUC) and cut-off values of corneal volume (CV) for the 3 mm, 5 mm, and 7 mm zones for distinguishing Group A (*n* = 198) from Group B2 (*n* = 206).

	AUC	SE	*p*	Cut-off value (D)	Sensitivity (%)	Specificity (%)
CV of 3 mm zone	0.925	0.014	<0.001	0.752	84.3	90.9
CV of 5 mm zone	0.883	0.017	<0.001	0.644	77.0	87.4
CV of 7 mm zone	0.842	0.020	<0.001	0.573	70.1	85.4

**Table 7:** Area under the curve (AUC) and cut-off values of corneal volume (CV) for the 3 mm, 5 mm, and 7 mm zones for distinguishing Group A (*n* = 198) from Group B3 (*n* = 140).

	AUC	SE	*p*	Cut-off value (D)	Sensitivity (%)	Specificity (%)
CV of 3 mm zone	0.953	0.012	<0.001	0.802	89.3	90.9
CV of 5 mm zone	0.883	0.018	<0.001	0.617	81.4	80.3
CV of 7 mm zone	0.794	0.025	<0.001	0.453	72.1	73.2

## Data Availability

The data that support the findings of this study are available on request from the corresponding author upon reasonable request.
